# The Effects of Shoe Sole Thickness on Running Style and Stability During Downhill Running at Different Speeds

**DOI:** 10.1002/ejsc.70116

**Published:** 2025-12-23

**Authors:** Cagla Kettner, Bernd J. Stetter, Thorsten Stein

**Affiliations:** ^1^ BioMotion Center Institute of Sports and Sports Science Karlsruhe Institute of Technology (KIT) Karlsruhe Germany; ^2^ Sports Orthopedics Institute of Sports and Sports Science Karlsruhe Institute of Technology (KIT) Karlsruhe Germany

**Keywords:** advanced footwear technologies (AFT), nonlinear analysis, sloped running, stack height

## Abstract

Advanced footwear technologies (AFT) like carbon plates and thick, lightweight soles were developed to enhance running performance. Previous research on sole thickness focused on level running; however, downhill running, with different biomechanical demands, remains underexplored. This study investigates how running shoe sole thickness affects running style and stability during downhill running at different speeds. Seventeen experienced male runners ran at 10 and 15 km/h on a −10% slope in three shoe conditions: a traditional control shoe (CON27, 27 mm), a thinner AFT‐shoe (AFT35, 35 mm), and a thicker AFT‐shoe (AFT50, 50 mm). Running style was analyzed using step frequency normalized to leg length, duty factor, vertical center of mass oscillation, vertical stiffness, leg stiffness, and lower limb angles in the sagittal and frontal planes. Increased stability was assessed using both nonlinear (lower maximum Lyapunov exponent for local stability and lower detrended fluctuation analysis for global stability) and linear methods (reduced ankle eversion for ankle stability). Both AFT35 and AFT50 altered running style via changes in ankle and knee kinematics (*p* = 0.001) and improved global stability (*p* = 0.004) compared to CON27 but did not affect spatiotemporal variables or local stability. Within AFT design, AFT50 affected ankle kinematics in both the sagittal and frontal planes, with differences of up to ∼4° (*p* < 0.001). These effects were consistent across running speeds. In conclusion, AFT‐shoes characterized with thicker soles influence joint kinematics and global stability during downhill running, whereas sole thickness within AFT designs primarily affects ankle stability and sagittal kinematics.

## Highlights

- During downhill running, AFT‐shoes with 35 and 50 mm sole thickness modulate ankle and knee kinematics as well as global stability compared to a traditional shoe without changing basic spatiotemporal variables or local stability.
- Within AFT designs, a thicker sole (50 mm) affects ankle kinematics compared to a thinner sole (35 mm), with no effects on other lower limb joint kinematics, spatiotemporal variables, global or local stability.
- These biomechanical and stability effects remain consistent across running speeds.

## 1 Introduction

In the 2010s, maximalist running shoes emerged, benefiting from Advanced Footwear Technologies (AFTs) to enhance running performance (Bermon et al. 2021; Rodrigo‐Carranza et al. 2021). AFT‐shoes are characterized by various technological advancements, including carbon plates or rods designed to optimize shoe bending stiffness and minimize mechanical energy loss at the foot joints (Ortega et al. 2021; Rodrigo‐Carranza et al. 2022). These carbon elements are typically embedded in a lightweight, thick midsole foam that provides both cushioning and energy return (Hoogkamer et al. 2018). In response to these technological changes, World Athletics introduced regulations limiting shoe sole thickness and restricting carbon plate use to a single element (World Athletics Council 2022). However, the biomechanical effects of sole thickness itself remain debated. Some studies suggest thicker soles may enhance performance by increasing effective leg length and stride length (Burns and Tam 2020; Ruiz‐Alias et al. 2023), whereas others attribute benefits mainly to foam and plate interactions (Bertschy et al. 2023, 2025). There are also concerns that thicker soles could impair ankle stability and elevate injury risk (Barrons et al. 2023; Hoogkamer 2020). A recent systematic review of sole thickness effects reported that thicker soles increase stance time and ankle dorsiflexion at initial contact, but have inconsistent effects on sagittal plane knee, hip, as well as frontal plane ankle kinematics (Kettner et al. 2025). Importantly, their influence on running style and stability has not been fully investigated.

Running style is considered important for both performance enhancement and injury prevention (Barnes and Kilding 2015; Folland et al. 2017; Saunders et al. 2004). van Oeveren et al. (2021) proposed a dual‐axis framework based on duty factor (DF) and step frequency normalized to leg length (SF_norm_) to operationalize running style. This approach is simple and intuitive, and it has been widely applied in both research and coaching to distinguish observable styles such as ‘small‐step’ and ‘large‐step’ running and to quantify modulations between conditions (e.g., different shoes; Kettner et al. 2025a; Nijs et al. 2023; Van der Meulen et al. 2024). However, these spatiotemporal variables do not fully capture underlying biomechanical differences (B. Hunter et al. 2023; Koegel et al. 2024; Mo et al. 2020; TenBroek et al. 2014). For example, changes in sole thickness can alter joint kinematics, particularly in the knee, and ankle during landing, and midstance (TenBroek et al. 2014). Recent studies also suggest that sole thickness can influence variables such as leg stiffness, and vertical center of mass oscillation (COM_osc_), pointing toward broader effects on running style (Kettner et al. 2025a).

Running stability is another critical aspect related to both running performance and the risk of injuries (Frank et al. 2019; Hoenig et al. 2019; Promsri et al. 2024; Schütte et al. 2018). Lower‐limb asymmetries, which commonly arise as functional adaptations to limb dominance and long‐standing sport participation and are observable even during cyclic tasks such as running (Maloney 2018), illustrate that runners naturally manage inter‐limb differences. This potentially increases the demands placed on stability control and underscores the relevance of evaluating stability from a broader motor‐control perspective. In biomechanics, stability refers to the ability to maintain the movement despite a perturbation (Stergiou 2016). Stability is typically classified into three categories: local, orbital, or global (Dingwell and Kang 2007). This study focuses specifically on local and global stability, as these are the most relevant and commonly analyzed in the context of running (B. Hunter et al. 2023). Both types are generally assessed using nonlinear analysis methods. Local dynamic stability is typically operationalized by the maximum Lyapunov exponent (MLE), which has shown sensitivity to factors like fatigue, running speed, and skill level (Frank et al. 2019; Hoenig et al. 2019; Mehdizadeh et al. 2014). Although studied less frequently, global stability can be assessed using detrended fluctuation analysis (DFA) of stride intervals. In this context, increased long‐range correlations are interpreted as reduced adaptability, indicating lower global stability (Agresta et al. 2019; Fuller et al. 2016; Hausdorff et al. 1996). In addition to nonlinear analysis methods, linear measures such as frontal plane ankle motion have been used to assess ankle stability. Variables like the peak eversion angle and duration have been linked to injury risk (Barrons et al. 2023; Becker et al. 2017; Hannigan and Pollard 2020). For example, Barrons et al. (2023) reported increased peak eversion wearing shoes with thicker soles (45 vs. 35 mm), suggesting reduced ankle stability for thicker soles. As most studies focus exclusively on either linear or nonlinear approaches, an integrated understanding of running stability across these domains remains limited (Barrons et al. 2023; Kettner et al. 2025a; Law et al. 2019).

Importantly, almost all previous work on sole thickness effects has investigated level running, with no studies specifically addressing downhill conditions (Kettner et al. 2025). There are a few studies investigating running shoe effects during downhill running, but no study focuses particularly on sole thickness effects (Chan et al. 2018; I. Hunter et al. 2022; Lu et al. 2024; Lussiana et al. 2016; Whiting et al. 2022). These studies either compared shoe models that differed in multiple critical shoe features (Chan et al. 2018; I. Hunter et al. 2022; Lussiana et al. 2016; Whiting et al. 2022) or focused on bending stiffness (Lu et al. 2024). This is a critical gap, since downhill running imposes distinct biomechanical and physiological demands. Unlike level running, downhill running requires the lower limb muscles to absorb energy through prolonged eccentric contractions, which increases muscle damage, soreness, and fatigue and can impair performance for several days (Bontemps et al. 2020). It also relies on mechanical energy dissipation rather than elastic storage and return, so shoe features that are advantageous in level running (e.g., highly resilient foams) may provide less benefit or even become detrimental (Whiting et al. 2022). From a performance perspective, downhill sections are decisive in trail running races (Genitrini et al. 2022), further highlighting their practical importance. Together, these factors demonstrate that downhill running is not simply a variant of level running, but can be considered a unique biomechanical case that requires dedicated investigation. Because downhill running requires increased eccentric braking and heightened demands on frontal‐plane stability due to increased joint moments, sole thickness may have a particularly strong influence on joint kinematics and stability responses under these conditions. Examining the influence of sole thickness under these conditions may provide important insights for both performance optimization and injury prevention in the future.

To address this research gap, the present study investigated the effects of different shoe sole thicknesses on running style and stability at different speeds. Three hypotheses were proposed. First, it was hypothesized that AFT‐shoes with thicker soles (50 and 35 mm) would modulate running style and reduce both local and global stability compared to a traditional shoe with a thinner sole (27 mm). Second, the AFT‐shoe with the thickest sole (50 mm) would lead to changes in running style and reduce both local and global stability compared to the AFT‐shoe with a thinner sole (35 mm). Third, these shoe‐related effects would be more pronounced at a higher running speed.

## 2 Methods

### 2.1 Participants

Seventeen healthy, experienced male runners participated in the study (mean age: 25.7 ± 3.9 years); height: 1.77 ± 0.04 m; body mass: 68.1 ± 6.0 kg; shoe size: EU 42–43; weekly running frequency: 4.2 ± 1.8 days; weekly distance: 33.7 ± 22.4 km). Downhill running experience was not specifically controlled or quantified; participants were included based on general running experience only. Sample size was determined based on related studies with a similar design (two within‐subject factors, one being shoe (TenBroek et al. 2013; Vercruyssen et al. 2016; Weir et al. 2020), and on medium‐sized effects (effect size, *f* = 0.25; power 80%; at significance level, *α* = 0.05) calculated using G*Power 3.1.9.7. All participants provided written informed consent. The study was approved by the Ethics Committee of the Karlsruhe Institute of Technology.

### 2.2 Measurement Protocol

Measurements were conducted on a motorized treadmill (h/*p*/cosmos Saturn, Nussdorf‐Traunstein, Germany). Participants began with a 5‐min treadmill familiarization session, running at a self‐selected speed in their own shoes across three slope conditions: level, downhill (−10%), and uphill (+10%). Following familiarization, all participants ran wearing three standardized shoes in a parallelized order via separate measurement blocks for each. The test shoes differed in sole thickness: 50 mm (AFT50), 35 mm (AFT35), and 27 mm (CON27), measured at the heel, US men's size 9. All available shoe specifications are reported in Table 1. Sole thickness was measured at the heel (75% of internal shoe length) and forefoot (12% of internal shoe length) in accordance with World Athletics regulations (World Athletics Council 2022). AFT50 and AFT35 were advanced footwear technology models with curved carbon‐infused rods (Barrons et al. 2023), whereas CON27 was a traditional model (Adidas Adizero RC4) with only TORSIONRODS. The two AFT‐shoes were selected to represent the lower and upper ends of typical AFT designs. The 50‐mm model exceeds the current World Athletics limit (World Athletics Council 2022) for road racing, but similar sole thicknesses are available in commercial shoes and are common in trail running shoes, which are not subject to this restriction. Trail‐specific shoes were not included here because the study was conducted under controlled treadmill conditions using road shoes. The CON27 shoe was included as a reference condition, chosen to closely match the AFT models in mass (maximum difference < 50 g) and heel‐to‐toe drop (7–8 mm), thereby minimizing potential confounding factors. Apart from sole thickness, the AFT35 and AFT50 shoes were identical in their design, which allowed us to specifically test the influence of sole thickness on running style and stability.

**TABLE 1:** Shoe specifications of the tested running shoes.

	Shoe type		
	AFT50	AFT35	CON27
Forefoot height (mm)	43	28	19
Heel height (mm)	50	35	27
Heel‐to‐toe drop (mm)	7	7	8
Mass (g)	268	220	219

Each measurement block included a 5‐min familiarization (Huang et al. 2022) with the current shoe of a 3‐min run and a 2‐min walk at self‐selected speed under level conditions. This was followed by three slope conditions: level, downhill (−10%), and uphill (+10%). This study focused exclusively on the downhill condition (−10%) because each slope condition represents a distinct motor task with unique biomechanical and energetic demands (Bontemps et al. 2020; Whiting et al. 2022), and running speeds were not metabolically matched across slopes (I. Hunter et al. 2022). The results for level running have been presented in previous publications (Kettner et al. 2025b, 2025a). Downhill running trials were conducted at 10 and 15 km/h with a −10% slope, which were selected based on previous research and pilot testing (Bontemps et al. 2020; Fadillioglu et al. 2022; I. Hunter et al. 2022). Participants ran for 25–40 s before each trial to allow the treadmill to reach the target speed, and then data were recorded for 90 s. To minimize fatigue, 1‐min walking breaks were provided between slow and fast runs, 2‐min standing breaks between slope conditions, and 5‐min sitting breaks between shoes. Perceived exertion was controlled using the Borg scale ([6–20]; Borg 1982), and trials only proceeded if the participant's rating was ≤ 12.

### 2.3 Data Acquisition, Data Processing, and Biomechanical Modeling

A 3D motion capture system (Vicon Motion Systems, Oxford, UK; 200 Hz) with 65 reflective markers was used to record full‐body kinematics. Reflective markers were placed on anatomical landmarks of the head, trunk, pelvis, and upper and lower extremities, following a combined ALASKA/Dynamicus (Hermsdorf et al. 2019) and Vicon Plug‐in Gait (Vicon Motion Systems Ltd 2023) protocols, with additional four‐marker clusters on the thigh and shank to reduce soft‐tissue artifacts (Ji et al. 2023). Marker trajectories were reconstructed offline using Vicon Nexus V2.12, and further data processing was performed in MATLAB (2024a, MathWorks Inc., Natick, MA, USA). The marker data were filtered using a fourth‐order low‐pass Butterworth filter with a 10‐Hz cutoff frequency (Gullstrand et al. 2009).

Inverse kinematics was computed using a modified version of the OpenSim Hamner Running Model (Hamner et al. 2010) to obtain the joint angles and COM. This model was originally developed for level running (Hamner et al. 2010) and has been widely applied in running biomechanics research (Hamner and Delp 2013; Kettner et al. 2025b; Nitschke et al. 2020). Although not specifically validated for downhill running, its use in this study was considered appropriate because whole‐body kinematics were the primary outcomes. It was therefore assumed that the model's kinematic estimates remain valid under the downhill slope condition used here. Each model was individually scaled until the maximum marker error was below 2 cm and the root mean square error was under 1 cm, using equal weights for all markers.

Initial contact events were identified by the local minimum in the mean of heel and toe marker velocities (Leitch et al. 2011), whereas toe‐off events were determined based on the maximum sagittal knee extension (Fellin et al. 2010). The used event‐detection algorithms were validated for treadmill level running with minimal timing errors (< 20 ms; Fellin et al. 2010; King et al. 2019; Leitch et al. 2011), and additional testing with 10 healthy adults during downhill running confirmed that errors remained within this range.

Linear and nonlinear analyses were performed using 25 and 100 left‐leg strides, respectively (Riazati et al. 2019; Winter et al. 2024). Strides from one leg, rather than both, were chosen in accordance with the methodology commonly used in running shoe and biomechanics studies (Kettner et al. 2025). Strides for linear analysis were selected from ∼15%–35% of the 90‐s trial, whereas those for nonlinear analysis were taken from ∼5%–85% of the trial duration.

### 2.4 Data Analysis

In line with the study rationale, critical variables were defined a priori. For running style, these were DF and SF_norm_ based on the dual‐axis framework (van Oeveren et al. 2021), and the sagittal and frontal hip, knee, and ankle angles. Complementary measures included COM_osc_, and stiffness variables. For running stability, MLE was defined as the critical measure of local stability and DFA as the critical measure of global stability (B. Hunter et al. 2023; Jordan et al. 2009). Linear ankle stability variables were included as complementary outcomes because they are frequently reported in the literature (Barrons et al. 2023; Kettner et al. 2025a; TenBroek et al. 2014). Complementary measures were analyzed to provide additional context but were not central to the hypotheses.

### 2.5 Running Style

#### 2.5.1 Spatiotemporal Variables

The SF_norm_ and DF were calculated based on the dual‐axis framework for running style (van Oeveren et al. 2021). Within this framework, lower DF indicates relatively shorter ground contact and higher flight time, whereas higher DF reflects longer ground contact. Likewise, SF_norm_ represents the cadence‐step‐length trade‐off at a given speed, where lower values correspond to longer steps and higher values correspond to shorter steps. The step frequency was calculated using initial contacts and normalized to leg length (*l*_0,_ the great trochanter to ground distance in a standing position; Morin et al. 2005) to get SF_norm_, where *g* is gravitational acceleration (Equation 1; Hof 1996; van Oeveren et al. 2021).

(1) $${\text{SF}}_{\text{norm}}=\frac{\text{SF}}{\sqrt{\frac{g}{l_{0}}}}$$

The DF was calculated as the ratio of stance time to twice the sum of stance and flight time (Equation 2; van Oeveren et al. 2021).

(2) $$\text{DF}=\frac{t_{\text{stance}}}{2\;\left(t_{\text{stance}}+t_{\text{flight}}\right)}$$

#### 2.5.2 Vertical Center of Mass Oscillation and Stiffness

COM_osc_ was calculated as the vertical range of COM movement during stance. k_ver_ and k_leg_ were estimated using Equations (3) and (4), respectively, where *m* is the body mass and v is the running speed (Morin et al. 2005).

(3) $$k_{\text{ver}}=\frac{m\;g\;\frac{\text{π}}{2}\;\left(\frac{t_{\text{flight}}}{t_{\text{stance}}}+1\right)}{\text{COMosc}}$$

(4) $$k_{\text{leg}}=\frac{m\;g\frac{\text{π}}{\;2}\;\left(\frac{t_{\text{flight}}}{t_{\text{stance}}}+1\right)}{l_{0}\;–\;\sqrt{{l_{0}}^{2}-{\left(\frac{v\;t_{\text{stance}}}{2}\right)}^{2}}+\text{COMosc}}$$

#### 2.5.3 Joint Kinematics

Frontal and sagittal joint angle time series for the ankle, knee, and hip were time‐normalized to the stance phase (101 points per cycle) (Kettner et al. 2025a; Möhler et al. 2022).

### 2.6 Running Stability

#### 2.6.1 Local Stability

Local dynamic stability of the foot, hip, trunk, and head was quantified using the MLE of marker clusters (4 markers per region; Ekizos et al. 2018; B. Hunter et al. 2023; Winter et al. 2024). Time series from 100 strides were normalized to 10,000 points (Hoenig et al. 2019; Raffalt et al. 2019). The embedding dimension (*m*) was determined using the false nearest neighbor method (Stergiou 2016; Wallot and Mønster 2018), and the highest *m* value across all trials was used per region (*m*_head_ = 9; *m*_trunk_ = 9; *m*_hip_ = 8; *m*_foot_ = 10). Time delay (*τ*) was based on the first local minimum of average mutual information curves, and the median *τ* value across all trials was used per region (*τ*_head_ = 22; *τ*_trunk_ = 23; *τ*_hip_ = 23; *τ*_foot_ = 33; Hoenig et al. 2019). MLE was calculated using Rosenstein's algorithm (Equation 5; Rosenstein et al. 1993), with the short‐term slope (*λ*) representing local stability. A lower *λ* indicated a higher dynamic local stability and vice versa.

(5) S(t)=[z(t),z(t+τ),…,z(t+(m−1)τ)]

#### 2.6.2 Global Stability

DFA was used to evaluate long‐range stride time correlations (B. Hunter et al. 2023; Jordan et al. 2009). Stride time series x were mean‐centered, cumulatively summed (Equation 6), and segmented into nonoverlapping windows of Δn = 4–24 points. The choice of this range followed recommendations for short stride series (≤ 128 data points) by Phinyomark et al. (2020). Within each segment, a second‐order polynomial trend was removed to account for potential low‐frequency drifts, and the root mean square fluctuation was calculated (Equation 7; Bryce and Sprague 2012).

(6) $$A\left(w\right)=\sum_{i=1}^{w}\left(x\left(i\right)-x_{avg}\right)$$

(7) $$F\left(\Delta n\right)=\sqrt{\frac{1}{N}\;\sum_{w=1}^{N}{\left(A\left(w\right)-A_{\Delta n}\left(w\right)\right)}^{2}}$$

If the data follow a power‐law, a log–log plot of F(Δn) versus Δn would yield a linear relationship (Equation 8). The slope (DFA‐α) quantifies correlation strength, where values between 0.5 and 1 indicate persistent behavior (Agresta et al. 2019; Jordan et al. 2009). In this range, a greater DFA‐α indicates a reduced global stability, and vice versa.

(8) $$F\;\left(\Delta n\right)\propto {\left(\Delta n\right)}^{\text{DFA}-\alpha }$$

#### 2.6.3 Ankle Stability

The linear measures peak eversion (MAX_eversion_) and eversion duration (t_eversion_) during stance were calculated to assess ankle stability (Barrons et al. 2023; Hannigan and Pollard 2020; Kettner et al. 2025a; TenBroek et al. 2014).

### 2.7 Statistics

Statistical analyses for discrete variables were conducted in SPSS (Version 29.0, IBM, Armonk, NY, USA). For linear analysis, means across 25 cycles per trial were used; for nonlinear analysis, one value per 100‐cycle trial was obtained. Normality and sphericity were assessed using Kolmogorov–Smirnov and Mauchly's tests, respectively. Greenhouse–Geisser corrections were applied where sphericity was violated. Repeated‐measures ANOVAs (rmANOVAs) were performed to compare shoe (AFT50, AFT35, CON27) and speed (10 km/h, 15 km/h) conditions. Effect sizes were estimated using partial eta squared ($\eta_{p}^{2}$: small ≤ 0.06, medium 0.06–0.14, large ≥ 0.14). *Post hoc* paired *t*‐tests with Bonferroni–Holm corrections were used in case of significant main shoe effects. Thereby, mean values across speeds were compared. Effect sizes for *post hoc* comparisons were calculated with Cohen's *d* (small ≤ 0.50, medium 0.50–0.80, large ≥ 0.80; Cohen 1988). Significance was set at *α* = 0.05.

Time‐series joint angle data were analyzed using statistical parametric mapping (SPM) in MATLAB (spm1d toolbox; Pataky et al. 2019). Normality was checked using the normality tests provided in the spm1d toolbox. In the case of nonnormality, nonparametric tests were performed with 1000 iterations. In case of significant shoe effects, *post hoc* paired *t*‐tests were performed. The significance level was set a priori to *α* = 0.05. For all SPM results reported, the cluster intervals are presented for descriptive purposes only. SPM inference applies to the trajectory as a whole rather than to isolated time points, so the exact cluster locations should not be overinterpreted (Honert and Pataky 2021).

Across all analyses, the main effects of shoe type and interaction effects between shoe and speed were further analyzed. However, the main effects of speed were not interpreted, as they were not directly related to our hypotheses. For completeness, results related to speed effects are reported in the tables.

## 3 Results

### 3.1 Running Style

**FIGURE 1:**
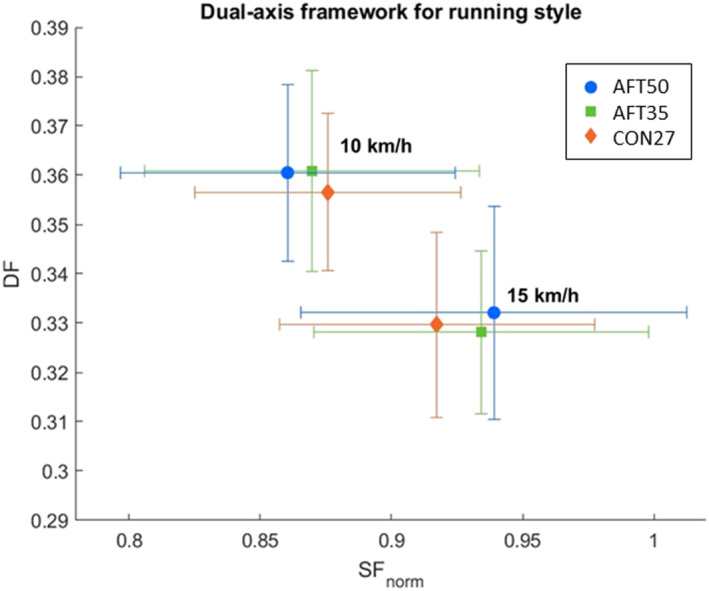
Running style analysis based on the dual‐axis framework (van Oeveren et al. 2021) across different shoe conditions [AFT50 (50 mm), AFT35 (35 mm), and CON27 (27 mm)] and running speeds (10 km/h and 15 km/h). Symbols represent mean values for each shoe, with error bars indicating standard deviations.

The results of the discrete parameters used in running style analysis are summarized in Table 2. Figure 1 illustrates the spatiotemporal variables (SF_norm_ and DF) within the dual‐axis framework for running style. COM_osc_ differed significantly between the shoes (*p* = 0.028). The *post hoc* results showed that AFT35 led to higher COM_osc_ compared to CON27(*p* = 0.006). AFT35 had a significantly (*p* = 0.005) lower k_ver_ than CON27.

**TABLE 2:** Discrete variables used for running style analysis include step frequency normalized to leg length (SF_norm_), duty factor (DF), vertical center of mass oscillation (COM_osc_), vertical stiffness (k_ver_), and leg stiffness (k_leg_).

	10 km/h	15 km/h	rmANOVA	F	*p*	$\eta_{p}^{2}$
SF_norm_						
AFT50	2.74 ± 0.20	2.96 ± 0.24	Shoe	1.54	0.235	0.09
AFT35	2.73 ± 0.20	2.91 ± 0.20	Speed	**57.00**	**< 0.001**	**0.78**
CON27	2.79 ± 0.15	2.98 ± 0.20	Interaction	1.06	0.345	0.06
DF						
AFT50	0.36 ± 0.02	0.33 ± 0.02	Shoe	2.52	0.097	0.14
AFT35	0.36 ± 0.02	0.33 ± 0.02	Speed	**58.35**	**< 0.001**	**0.79**
CON27	0.35 ± 0.02	0.33 ± 0.02	Interaction	0.82	0.448	0.05
COM_osc_ (m)						
AFT50	0.09 ± 0.01	0.09 ± 0.01	Shoe	**4.00**	**0.028**	**0.20**
AFT35	0.10 ± 0.01	0.09 ± 0.01	Speed	1.81	0.198	0.10
CON27	0.09 ± 0.01	0.09 ± 0.01	Interaction	1.93	0.161	0.11
			*Post hoc*	*t*	*p*	|d|
			AFT50‐AFT35	−1.54	0.141	0.38
			AFT50‐CON27	1.01	0.164	0.24
			AFT35‐CON27	**3.45**	**0.006**	**0.83**
k_ver_ (kN/m)						
AFT50	15.90 ± 2.93	17.97 ± 3.26	Shoe	**6.24**	**0.005**	**0.28**
AFT35	15.55 ± 2.82	17.49 ± 2.83	Speed	**70.85**	**< 0.001**	**0.82**
CON27	16.37 ± 2.64	18.12 ± 3.19	Interaction	0.68	0.513	0.04
			*Post hoc*	*t*	*p*	|d|
			AFT50‐AFT35	1.87	0.080	0.45
			AFT50‐CON27	−1.54	0.071	0.37
			AFT35‐CON27	**−3.76**	**0.002**	**0.91**
k_leg_ (kN/m)						
	10 km/h	15 km/h	rmANOVA	F	*p*	$\eta_{p}^{2}$
AFT50	8.90 ± 1.52	7.56 ± 1.12	Shoe	3.59	0.064	0.18
AFT35	8.60 ± 1.22	7.38 ± 1.06	Speed	**72.81**	**< 0.001**	**0.82**
CON27	9.25 ± 1.49	7.76 ± 1.24	Interaction	1.68	0.202	0.10

*Note:* Shoe sole thicknesses are abbreviated as AFT50 (50 mm), AFT35 (35 mm), and CON27 (27 mm). Descriptive statistics are presented as mean ± standard deviation. *Post hoc* comparisons were performed on mean data at 10 km/h and 15 km/h. Statistically significant results are indicated in bold.

**FIGURE 2:**
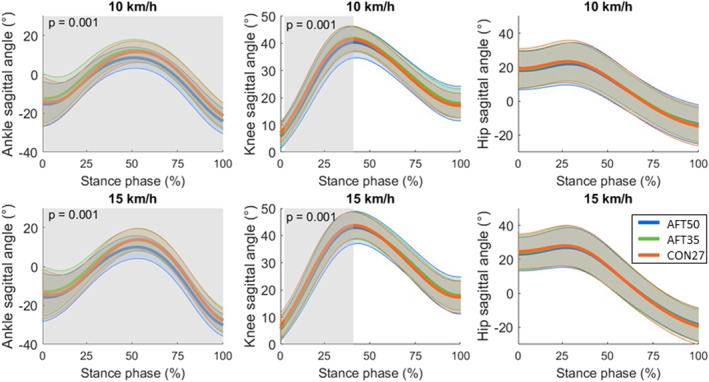
Sagittal plane joint angle time series for the ankle, knee, and hip, shown as mean trajectories (thick lines) ± standard deviation (thin lines). By convention, positive angles indicate joint flexion (dorsiflexion for the ankle). Shaded regions highlight significant differences between shoes, independent of running speed, with corresponding cluster *p*‐values displayed. Shoe sole thicknesses are abbreviated as AFT50 (50 mm), AFT35 (35 mm), and CON27 (27 mm).

**FIGURE 3:**
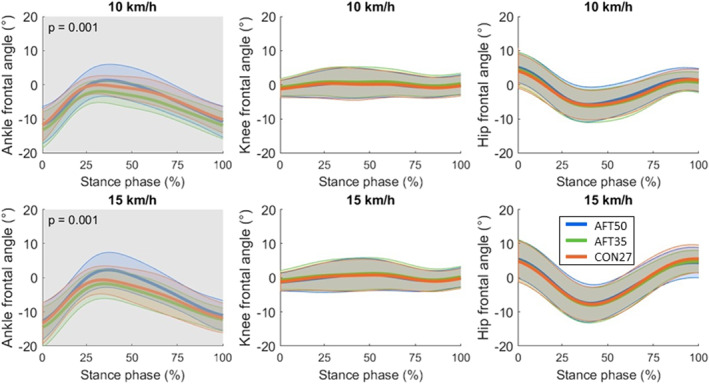
Frontal plane joint angle time series for the ankle, knee, and hip, shown as mean trajectories (thick lines) ± standard deviation (thin lines). By convention, positive angles indicate abduction at the knee and hip, and eversion at the ankle. Shaded regions indicate significant differences between shoes, independent of running speed, with corresponding cluster *p*‐values shown. Shoe sole thicknesses are abbreviated as AFT50 (50 mm), AFT35 (35 mm), and CON27 (27 mm).

Joint angle time‐series analyses are presented in Figures 2 and 3 for the sagittal and frontal planes, respectively. SPM results revealed significant shoe differences in the sagittal ankle angle (*p* = 0.001; cluster spanning 0%–100% of the stance phase; Figure 2). The *post hoc* tests showed that the AFT50 had less dorsiflexion compared to AFT35 (*p* < 0.001; cluster spanning 0%–100% of the stance phase) and CON27 (*p* < 0.001; cluster spanning 29%–100% of the stance phase) and AFT35 had greater dorsiflexion than CON27 (*p* = 0.001; cluster spanning 0%–45% of the stance phase; Figure 1 in Supporting Information S1). Sagittal knee angle also differed significantly between shoes (*p* = 0.001; cluster spanning 2%–38% of the stance phase; Figure 2), with greater knee flexion in AFT35 than CON27 (*p* = 0.008; cluster spanning 64%–91% of the stance phase; Figure 2 in Supporting Information S1).

In the frontal plane, only the ankle showed significant shoe differences (*p* = 0.001; cluster spanning 0%–100% of the stance phase; Figure 3), with AFT50 having greater eversion than AFT35 (*p* < 0.001; cluster spanning 0%–100% of the stance phase) and CON27 (*p* = 0.002, cluster spanning 26%–61% of the stance phase; Figure 3 in Supporting Information S1).

### 3.2 Running Stability

Table 3 summarizes the results of all running stability variables. Significant differences were found in DFA‐α across shoe conditions (*p* = 0.004). DFA‐α was lower for AFT‐shoes (AFT50 vs. CON27, *p* = 0.006; AFT35 vs. CON27, *p* = 0.041). MAX_eversion_ also differed significantly between shoes (*p* < 0.001), with AFT50 showing the greatest eversion and AFT35 the least. Similarly, t_eversion_ showed significant differences (*p* < 0.001), with all pairwise comparisons being significant. AFT50 resulted in the longest eversion duration, whereas AFT35 showed the shortest.

**TABLE 3:** Variables used for running stability analysis include local dynamic stability of the head (*λ*_head_), trunk (*λ*_trunk_), hip (*λ*_hip_), and foot (*λ*_foot_), detrended fluctuation scaling component of stride time (DFA‐α), peak eversion (MAX_eversion_), and eversion duration (t_ieversion_) during stance.

	10 km/h	15 km/h	rmANOVA	F	*p*	$\eta_{p}^{2}$
*λ*_head_						
AFT50	1.31 ± 0.13	1.35 ± 0.11	Shoe	0.53	0.591	0.03
AFT35	1.27 ± 0.11	1.38 ± 0.08	Speed	3.85	0.067	0.19
CON27	1.31 ± 0.12	1.32 ± 0.10	Interaction	3.09	0.059	0.16
*λ*_trunk_						
AFT50	1.26 ± 0.11	1.25 ± 0.15	Shoe	2.73	0.081	0.15
AFT35	1.24 ± 0.12	1.31 ± 0.11	Speed	0.70	0.416	0.04
CON27	1.22 ± 0.10	1.22 ± 0.10	Interaction	1.60	0.218	0.09
*λ*_hip_						
AFT50	1.42 ± 0.11	1.43 ± 0.13	Shoe	0.08	0.920	0.01
AFT35	1.39 ± 0.14	1.46 ± 0.11	Speed	1.51	0.236	0.09
CON27	1.42 ± 0.13	1.45 ± 0.12	Interaction	1.09	0.350	0.06
*λ*_foot_						
AFT50	0.74 ± 0.09	0.81 ± 0.10	Shoe	0.14	0.875	0.01
AFT35	0.73 ± 0.12	0.80 ± 0.14	Speed	11.24	**0.004**	**0.41**
CON27	0.72 ± 0.13	0.81 ± 0.12	Interaction	0.22	0.806	0.01
DFA‐α						
AFT50	0.57 ± 0.14	0.69 ± 0.12	Shoe	**6.59**	**0.004**	**0.29**
AFT35	0.61 ± 0.14	0.73 ± 0.16	Speed	**10.43**	**0.005**	**0.40**
CON27	0.68 ± 0.18	0.76 ± 0.15	Interaction	0.32	0.731	0.02
			*Post hoc*	*t*	*p*	|d|
			AFT50‐AFT35	−1.93	0.072	0.47
			AFT50‐CON27	**−3.41**	**0.006**	**0.83**
			AFT35‐CON27	**−1.86**	**0.041**	**0.45**
MAX_eversion_ (°)						
AFT50	1.71 ± 4.82	2.70 ± 5.17	Shoe	**27.33**	**< 0.001**	**0.63**
AFT35	−1.58 ± 3.37	−1.46 ± 4.32	Speed	0.40	0.539	0.02
CON27	0.28 ± 2.70	−0.20 ± 3.89	Interaction	2.05	0.146	0.11
			*Post hoc*	*t*	*p*	|d|
			AFT50‐AFT35	**7.35**	**< 0.001**	**1.78**
			AFT50‐CON27	**3.40**	**0.002**	**0.82**
			AFT35‐CON27	**−4.79**	**< 0.001**	**1.16**
t_eversion_ (%)						
AFT50	24.05 ± 24.08	24.73 ± 21.92	Shoe	**0.01**	**< 0.001**	**0.44**
AFT35	9.51 ± 17.02	9.97 ± 18.99	Speed	12.65	0.929	0.01
CON27	16.94 ± 21.87	16.20 ± 19.81	Interaction	0.11	0.893	0.07
			*Post hoc*	t	*p*	|d|
			AFT50‐AFT35	**4.39**	**< 0.001**	**1.06**
			AFT50‐CON27	**2.29**	**0.018**	**0.55**
			AFT35‐CON27	**−4.19**	**< 0.001**	**1.02**

*Note:* Shoe sole thicknesses are abbreviated as AFT50 (50 mm), AFT35 (35 mm), and CON27 (27 mm). Descriptive statistics are reported as mean ± standard deviation. *Post hoc* analyses were conducted on mean data at 10 km/h and 15 km/h. Statistically significant results are indicated in bold.

## 4 Discussion

This study investigated the effects of different sole thicknesses on running style and stability during downhill running at different speeds. Consistent with the first hypothesis, thicker‐soled AFT shoes (AFT50 and AFT35) modified ankle and knee angles. However, contrary to the first hypothesis, they enhanced global stability compared with a traditional shoe (CON27), while spatiotemporal variables and local stability remained unaffected. Partially supporting the second hypothesis, increasing sole thickness from 35 to 50 mm specifically increased plantarflexion and eversion without altering other lower limb joints kinematics, spatiotemporal variables, or stability outcomes. Contrary to the third hypothesis, these effects were consistent across both tested running speeds.

### 4.1 AFT‐Shoes Altered Joint Kinematics Without Affecting Spatiotemporal Variables

The first hypothesis proposed that the AFT‐shoes with thicker soles would lead to changes in running style compared to a relatively thinner, traditional shoe. The results provided partial support for this hypothesis. Specifically, spatiotemporal variables (SF_norm_ and DF) were not affected by sole thickness, indicating that fundamental aspects of running style remained stable across shoe conditions. This is consistent with spring‐mass model in which the spatiotemporal structure of running (e.g., characterized by stride frequency and the ratio between stance and flight phases) is determined by the spring‐like leg behavior at a given running speed (Blickhan 1989; van Oeveren et al. 2021). By contrast, downhill running relies on eccentric braking and energy dissipation (Bontemps et al. 2020; Gottschall and Kram 2005; Whiting et al. 2022), making joint kinematics possibly more sensitive to shoe properties even when DF and SF_norm_ remain unchanged. Moreover, multivariate cluster analysis in a previous study has shown that variables such as leg stiffness and COM oscillation differentiate running patterns more strongly than spatiotemporal metrics alone (Koegel et al. 2024). COM_osc_ was slightly higher with the thinner AFT‐shoe compared to the traditional shoe, although the difference in mean values was less than 1 cm. This increase was accompanied by a decrease in k_ver_, which is inversely proportional to COM_osc_ (Equation 3). Joint kinematics revealed shoe‐related differences, particularly at the ankle. SPM analysis showed significant differences across the stance trajectory, but the timing of threshold crossings should not be taken as proof of phase‐specific effects. The cluster locations are shown only to help interpret them within running phases, since the statistical inference in SPM is made on the full time‐normalized trajectory (Honert and Pataky 2021). The thicker AFT‐shoe resulted in reduced dorsiflexion and increased eversion compared with both the thinner AFT‐shoe and traditional shoe. Conversely, thinner AFT‐shoe showed greater dorsiflexion and knee flexion than traditional shoes. The higher COM_osc_ observed in the thinner AFT‐shoe compared with the traditional shoe can be attributed to this increased dorsiflexion and knee flexion, which likely lowered the COM during stance (van Oeveren et al. 2021) and consequently increased the vertical range of COM motion.

In level running with the same experimental shoes (Kettner et al. 2025a), the thicker AFT‐shoes led to a reduced SF_norm_ compared to the traditional shoe at 15 km/h. The thicker AFT‐shoe led to greater DF than the traditional shoe across both speeds. Findings for COM_osc_ were consistent across downhill and level running. In level running, increasing sole thickness led to greater COM_osc_ and lower k_ver_ among all shoe comparisons. However, joint kinematic changes during level running were less pronounced, particularly in the frontal ankle plane, and no significant differences were observed at the knee. The differing results between downhill and level running can be attributed to the differing mechanical demands of each condition. Specifically, downhill running relies more on mechanical energy dissipation and eccentric muscle loading than on elastic energy storage and return (Bontemps et al. 2020; Whiting et al. 2022). As a result, joint movements were more sensitive to variations in shoe properties due to the increased braking demands and shock absorption required in downhill running (Gottschall and Kram 2005). In contrast, spatiotemporal variables, which are more closely related to the spring‐like behavior of the lower limb during running (Blickhan 1989), were less affected by changes in shoes. The findings suggested that alhough shoes influenced joint kinematics during downhill running, the overall running style, as indicated by DF and SF_norm_ (van Oeveren et al. 2021), remained unchanged.

It should be noted that running style in this study was defined using spatiotemporal variables (DF and SF_norm_), which differs from traditional footstrike‐based classifications (e.g., rearfoot, midfoot, forefoot; Altman and Davis 2012). The spatiotemporal framework reflects how runners distribute ground contact time relative to flight time and adjust step frequency at a given speed, providing a continuous description of running style (van Oeveren et al. 2021). Footstrike, by contrast, categorizes the initial contact pattern and does not directly capture characteristics of the entire running cycle. Although related, the two descriptors are not interchangeable. Footstrike categories are simple to observe and remain relevant in practice, but DF and SF_norm_ provide complementary insights into spatiotemporal behavior. Together, these descriptors may inform running technique modifications beyond footstrike alone.

### 4.2 AFT‐Shoes Enhanced Global Stability But Not Local Stability

According to the first hypothesis, it was expected that AFT‐shoes would reduce both local and global running stability compared to the relatively thinner, traditional shoe. However, the results were mixed. Global stability improved with the AFT‐shoes, whereas local stability remained unaffected. However, ankle stability decreased with the thicker AFT‐shoe.

Global stability was higher with AFT‐shoes. Specifically, DFA‐α decreased with AFT‐shoes, suggesting more stable stride time dynamics (Agresta et al. 2019; Jordan et al. 2009). In contrast, local stability of the head, trunk, hip, and foot was not affected by sole thickness based on nonlinear analysis (Winter et al. 2024). This suggests that AFT‐shoes compared to the thinner traditional shoe may promote a more regular long‐range organization of stride timing across multiple steps, without altering the ability to recover from small perturbations at the local segmental level.

In contrast, ankle stability was lower with the thicker AFT‐shoe compared to the traditional shoe, as indicated by the greater MAX_eversion_ (Barrons et al. 2023). Additionally, t_eversion_ was longer with these shoes. These findings are consistent with those observed during level running (Kettner et al. 2025a). However, it should be noted that frontal plane kinematics are more challenging to model accurately (Wouda et al. 2018). Therefore, the observed differences in ankle stability may be partially influenced by modeling artifacts and should be interpreted with caution.

Global and local stability findings should be considered alongside commonly used ankle kinematic measures. Linear analysis variables such as peak eversion and eversion duration or velocity reflect ankle motion during stance and are often linked to injury risk (Barrons et al. 2023; Becker et al. 2017; Kuhman et al. 2016). In contrast, MLE reflects how the system responds to small perturbations, and DFA describes the organization of stride timing across many steps (Frank et al. 2019; Fuller et al. 2016; B. Hunter et al. 2023; Jordan et al. 2009). This study used both linear (peak eversion and eversion duration) and nonlinear methods (MLE and DFA) to analyze movement stability. Generally, linear measures (such as the standard deviation of ankle movement) show how much individual steps deviate from the average. Large variations may indicate that the joint moves closer to potentially risky positions, where small perturbations could become critical and increase the risk of injury. However, linear analysis assumes that each step is independent of the others, which is a simplifying assumption to describe the system but does not reflect the true nature of human movement. In reality, walking and running involve continuous, interconnected adjustments, where past movements influence future ones. Nonlinear analysis accounts for this complexity by examining how the system behaves over time. It reveals underlying patterns of coordination, adaptability, and stability that linear measures cannot capture (Stergiou 2016). For example, the differing findings between local foot stability and frontal ankle mechanics do not contradict each other, but highlight how each method reflects different aspects of movement. These findings also support that local and global stability measure separate components of system behavior (Dingwell and Kang 2007). It is therefore important to clearly define what kind of stability is being operationalized and interpreted.

### 4.3 Sole Thickness Within AFT Designs Primarily Affects Ankle Kinematics

The second hypothesis stated that the thicker AFT‐shoe would change running style and reduce both local and global stability compared to the thinner AFT‐shoe. The results provided only limited support for this hypothesis. Specifically, the thicker AFT‐shoe led to the greatest MAX_eversion_, indicating reduced ankle stability (Barrons et al. 2023). It also reduced dorsiflexion and increased eversion during stance compared to the thinner AFT‐shoe with differences up to 4°. In contrast, stability metrics, COM movement, or stiffness variables were not different between the two AFT‐shoes. These findings suggest that the increased sole thickness in AFT‐shoes primarily influenced ankle kinematics, rather than vertical loading characteristics or global stability. Importantly, the observed differences between ankle stability and global stability should not be interpreted as contradictory outcomes. These metrics quantify distinct levels of locomotor control. Global stability, operationalized with DFA‐α, reflects stride‐to‐stride temporal organization and whole‐body adaptability of the running pattern and did not differ between AFT50 and AFT35. In contrast, local ankle stability, operationalized through frontal‐plane eversion kinematics, reflects joint‐specific mechanical control that is particularly sensitive to footwear geometry and frontal‐plane loading. Accordingly, greater ankle eversion was observed in AFT50 compared to AFT35. Thus, a thicker sole may alter ankle posture and local mechanical leverage without necessarily modulating the overall temporal structure of running gait. The combination of reduced ankle stability but unchanged global stability therefore indicates that sole thickness difference within AFT design primarily affects segment‐level mechanics (i.e., frontal‐plane ankle kinematics), whereas system‐level gait dynamics (i.e., DFA‐ *α*) remain unchanged across shoes.

A previous case study by Fritz et al. (2023) reported that increased midsole cushioning, which is typically associated with decreased shoe stiffness (Barrons et al. 2023), may result in a compensatory increase in leg stiffness. This biomechanical adaptation was suggested to facilitate energy dissipation in the lower limbs and potentially improve running economy, particularly for downhill running. In contrast, the findings of the present study did not support this, as k_leg_ remained consistent across all shoe conditions. However, it should be noted that the stiffness variables in the current study were estimated using kinematic data (Morin et al. 2005), which may limit the precision of these measurements.

### 4.4 Shoe Effects Were Consistent Across Running Speeds

In general, higher running speeds may impose more challenging task conditions (Santuz et al. 2020), which potentially amplify the influence of sole thickness on running style and stability. Therefore, the third hypothesis proposed that the effects of different sole thicknesses would become more pronounced at higher speeds. However, the results did not support this hypothesis, as the observed differences between shoes were consistent across both running speeds. These findings align with those reported for level running (Kettner et al. 2025a). This suggests that the effects of sole thickness on running style and stability reflect global adaptations that are preserved across varying speed conditions.

### 4.5 Relation to Traditional Biomechanical Variables

The present study included variables less commonly applied in running shoe research, such as nonlinear stability measures. These do not replace traditional measures like impact forces, pronation angle, or center of pressure excursion (Kettner et al. 2025; Law et al. 2019), but rather complement them by capturing spring‐mass model variables and stride‐to‐stride adaptations. Specifically, vertical stiffness and COM oscillation reflect how runners absorb impact and regulate vertical motion (Blickhan 1989; Struzik et al. 2021). These aspects become especially relevant during downhill running, where impact forces and braking demands are substantially higher, requiring greater eccentric control to manage the downward movement of the body. Nonlinear stability measures further highlight dynamic adaptations over multiple strides (Fuller et al. 2016; B. Hunter et al. 2023; Stergiou 2016; Winter et al. 2024), thereby complementing linear descriptive approaches. In this way, the selected variables broaden the interpretation of how sole thickness affects running style and stability.

### 4.6 Limitations

This study has some limitations that should be mentioned. First, although the primary difference between the shoes was sole thickness, other shoe features also varied. In particular, shoe masses differed slightly, with a maximum difference of 49 g (Table 1). Although previous research suggests that an added mass of 50 g does not impact running economy or spatiotemporal variables (Rodrigo‐Carranza et al. 2020), its potential effect on stability remains unclear. Second, the study was conducted on a treadmill at two constant running speeds. Although this controlled setting aligns with many previous studies (e.g., Barrons et al. 2023; Chambon et al. 2014; TenBroek et al. 2014), it may not fully reflect real‐world situations. Factors such as uneven terrain, fatigue, or higher speeds might amplify shoe‐related effects. Moreover, treadmill trials were performed with road‐running shoes to avoid confounding from traction or outsole design. Future work should investigate whether the findings apply to trail conditions. Third, participants were healthy, experienced male runners, which limits the generalizability of results to other populations with different skill levels. This limitation is not unique to this study but it is a global research gap in similar studies. Moreover, the footstrike pattern was not controlled to preserve ecological validity (Kettner et al. 2025). Classifying the runners following (Altman and Davis 2012) revealed that 7 of 17 participants had a midfoot strike (5.1 ± 2.3°) and 10 of 17 a rearfoot strike (15.4 ± 2.8°), and none forefoot. Across shoe conditions, no significant changes in footstrike pattern were observed. Fourth, gait events (initial contact and toe‐off) were identified using kinematic data. Although the algorithms used were validated for treadmill use with minimal errors (≤ 20 ms; Fellin et al. 2010; King et al. 2019; Leitch et al. 2011), kinetic‐based methods are considered more accurate. Nevertheless, most analyzed variables were likely only minimally affected by this approach. Fifth, modeling movement in the frontal plane is more challenging than in the sagittal plane (Wouda et al. 2018). Furthermore, the full‐body model used in this study represents the foot as a single rigid segment and does not include a dedicated metatarsophalangeal joint or multisegment rearfoot/forefoot structure, which can decrease the accuracy of ankle joint kinematics (Wager and Challis 2024). Therefore, the observed differences in frontal ankle mechanics may partly reflect kinematic modeling artifacts and should be interpreted with caution. Furthermore, as only left‐leg strides were analyzed in line with similar running shoe studies (Kettner et al. 2025; Kettner et al. 2025a, 2025b), potential leg asymmetries cannot be excluded. Although bilateral differences in sagittal‐plane and spatiotemporal variables are typically small in healthy runners, greater asymmetry has been reported in frontal‐plane kinematics (Vincent et al. 2025), which may partly reflect limitations of current full‐body modeling approaches (Wouda et al. 2018). Finally, although shorter stride series are frequently used in DFA applications to gait variability (B. Hunter et al. 2023; Kuznetsov and Rhea 2017), some studies suggest that the recordings of ≤ 600 strides may not sufficiently capture long‐range correlations (Damouras et al. 2010). In this study, DFA parameters were selected in accordance with recommendations for short time series (Phinyomark et al. 2020). In addition, a sensitivity analysis with alternative box‐size ranges was conducted. Across the tested ranges, between‐shoe differences remained significant with large effect sizes.

## 5 Conclusion

This study investigated the effects of running shoe sole thickness on running style and stability during downhill running at different speeds. Two AFT models (50 and 35 mm) and a traditional control shoe (27 mm) were compared. Thicker‐soled AFT‐shoes altered ankle and knee kinematics and improved global stability relative to the traditional shoe, without influencing spatiotemporal variables or local stability. Within AFT‐shoes, increasing sole thickness from 35 to 50 mm primarily increased plantarflexion and eversion, with no further impact on other lower limb kinematics or stability outcomes. These effects were consistent across the two tested speeds. Future research should explore these human–shoe interactions under more variable conditions (e.g., fatigue), in more diverse populations (e.g., novice runners), and with experimental shoes that span larger stack‐height ranges (e.g., 10–50 mm) while minimizing remaining compounding factors such as plate stiffness, foam properties, and mass. Additionally, studies should investigate uphill running, which remains under researched and involves fundamentally different propulsive and clearance demands.

## Author Contributions

**Cagla Kettner:** writing – review and editing, writing – original draft, visualization, software, methodology, investigation, formal analysis, data curation, conceptualization. **Bernd J. Stetter:** writing – review and editing, conceptualization. **Thorsten Stein:** writing – review and editing, supervision, resources, project administration, methodology, funding acquisition, conceptualization.

## Funding

Adidas AG provided financial and material support for this study. The funder had no role in study design, data collection and analysis, decision to publish, or preparation of the manuscript.

## Ethics Statement

The study was approved by the Ethics Committee of the Karlsruhe Institute of Technology (KIT).

## Conflicts of Interest

The authors declare no conflicts of interest.

## Supporting information


**Supporting Information** S1.

## Data Availability

The raw data supporting the conclusions of this article will be made available by the authors upon reasonable request.

## References

[ejsc70116-bib-0001] Agresta, C. E. , G. C. Goulet , J. Peacock , J. Housner , R. F. Zernicke , and J. D. Zendler . 2019. “Years of Running Experience Influences Stride‐to‐Stride Fluctuations and Adaptive Response During Step Frequency Perturbations in Healthy Distance Runners.” Gait & Posture 70: 376–382. 10.1016/j.gaitpost.2019.02.034. 30959429 PMC7607923

[ejsc70116-bib-0002] Altman, A. R. , and I. S. Davis . 2012. “A Kinematic Method for Footstrike Pattern Detection in Barefoot and Shod Runners.” Gait & Posture 35, no. 2: 298–300. 10.1016/j.gaitpost.2011.09.104. 22075193 PMC3278526

[ejsc70116-bib-0003] Barnes, K. R. , and A. E. Kilding . 2015. “Running Economy: Measurement, Norms, and Determining Factors.” Sports Medicine ‐ Open 1, no. 1: 1–15. 10.1186/s40798-015-0007-y. 27747844 PMC4555089

[ejsc70116-bib-0004] Barrons, Z. B. , J. W. Wannop , and D. J. Stefanyshyn . 2023. “The Influence of Footwear Midsole Thickness on Running Economy and Frontal Plane Ankle Stability.” Footwear Science 15, no. 3: 155–160. 10.1080/19424280.2023.2218321.

[ejsc70116-bib-0005] Becker, J. , S. James , R. Wayner , L. Osternig , and L.‐S. Chou . 2017. “Biomechanical Factors Associated With Achilles Tendinopathy and Medial Tibial Stress Syndrome in Runners.” American Journal of Sports Medicine 45, no. 11: 2614–2621. 10.1177/0363546517708193. 28581815

[ejsc70116-bib-0006] Bermon, S. , F. Garrandes , A. Szabo , I. Berkovics , and P. E. Adami . 2021. “Effect of Advanced Shoe Technology on the Evolution of Road Race Times in Male and Female Elite Runners.” Frontiers in Sports and Active Living 3: 653173. 10.3389/fspor.2021.653173. 33969296 PMC8100054

[ejsc70116-bib-0007] Bertschy, M. , H. Lino , L. Healey , and W. Hoogkamer . 2023. “Effects of Midsole Stack Height and Foam on the Metabolic Cost of Running.” Supplement, Footwear Science 15, no. S1: S180–S181. 10.1080/19424280.2023.2202942.

[ejsc70116-bib-0008] Bertschy, M. , H. Lino , L. Healey , and W. Hoogkamer . 2025. “Is Increasing the Effective Leg Length of a Human Runner Metabolically Beneficial? Changing Material From Firm to.”. 10.1242/jeb.250107 PMC12582409 40955759

[ejsc70116-bib-0009] Blickhan, R. 1989. “The Spring‐Mass Model for Running and Hopping.” Journal of Biomechanics 22, no. 11: 1217–1227. 10.1016/0021-9290(89)90224-8. 2625422

[ejsc70116-bib-0010] Bontemps, B. , F. Vercruyssen , M. Gruet , and J. Louis . 2020. “Downhill Running: What Are the Effects and How Can We Adapt? A Narrative Review.” In Sports Medicine 50, no. 12: 2083–2110. Springer Science and Business Media Deutschland GmbH. 10.1007/s40279-020-01355-z 33037592 PMC7674385

[ejsc70116-bib-0011] Borg, G. A. V. 1982. “Psychophysical Bases of Perceived Exertion.” Medicine & Science in Sports & Exercise 14, no. 5: 377–381. https://journals.lww.com/acsm‐msse/fulltext/1982/05000/psychophysical_bases_of_perceived_exertion.12.aspx. 7154893

[ejsc70116-bib-0012] Bryce, R. M. , and K. B. Sprague . 2012. “Revisiting Detrended Fluctuation Analysis.” Scientific Reports 2, no. 1: 315. 10.1038/srep00315. 22419991 PMC3303145

[ejsc70116-bib-0013] Burns, G. T. , and N. Tam . 2020. “Is it the Shoes? A Simple Proposal for Regulating Footwear in Road Running.” British Journal of Sports Medicine 54, no. 8: 439–441. 10.1136/bjsports-2018-100480. 31630088

[ejsc70116-bib-0014] Chambon, N. , N. Delattre , N. Guéguen , E. Berton , and G. Rao . 2014. “Is Midsole Thickness a Key Parameter for the Running Pattern?” Gait & Posture 40, no. 1: 58–63. 10.1016/j.gaitpost.2014.02.005. 24636223

[ejsc70116-bib-0015] Chan, Z. Y. S. , I. P. H. Au , F. O. Y. Lau , E. C. K. Ching , J. H. Zhang , and R. T. H. Cheung . 2018. “Does Maximalist Footwear Lower Impact Loading During Level Ground and Downhill Running?” European Journal of Sport Science 18, no. 8: 1083–1089. 10.1080/17461391.2018.1472298. 29792108

[ejsc70116-bib-0016] Cohen, J. 1988. Statistical Power Analysis for the Behavioral Sciences. 2nd ed., 20–27 Lawrence Erlbaum Associates. 10.4324/9780203771587.

[ejsc70116-bib-0017] Damouras, S. , M. D. Chang , E. Sejdić , and T. Chau . 2010. “An Empirical Examination of Detrended Fluctuation Analysis for Gait Data.” Gait & Posture 31, no. 3: 336–340. 10.1016/j.gaitpost.2009.12.002. 20060298

[ejsc70116-bib-0018] Dingwell, J. B. , and H. G. Kang . 2007. “Differences Between Local and Orbital Dynamic Stability During Human Walking.” Journal of Biomechanical Engineering 129, no. 4: 586–593. 10.1115/1.2746383. 17655480

[ejsc70116-bib-0019] Ekizos, A. , A. Santuz , A. Schroll , and A. Arampatzis . 2018. “The Maximum Lyapunov Exponent During Walking and Running: Reliability Assessment of Different Marker‐Sets.” Frontiers in Physiology 9, (August): 1–11. 10.3389/fphys.2018.01101. 30197597 PMC6117405

[ejsc70116-bib-0020] Fadillioglu, C. , F. Möhler , M. Reuter , and T. Stein . 2022. “Changes in Key Biomechanical Parameters According to the Expertise Level in Runners at Different Running Speeds.” Bioengineering 9, no. 11: 616. 10.3390/bioengineering9110616. 36354527 PMC9687194

[ejsc70116-bib-0021] Fellin, R. E. , W. C. Rose , T. D. Royer , and I. S. Davis . 2010. “Comparison of Methods for Kinematic Identification of Footstrike and Toe‐Off During Overground and Treadmill Running.” Journal of Science and Medicine in Sport 13, no. 6: 646–650. 10.1016/j.jsams.2010.03.006. 20478742 PMC3266867

[ejsc70116-bib-0022] Folland, J. P. , S. J. Allen , M. I. Black , J. C. Handsaker , and S. E. Forrester . 2017. “Running Technique is an Important Component of Running Economy and Performance.” Medicine & Science in Sports & Exercise 49, no. 7: 1412–1423. 10.1249/MSS.0000000000001245. 28263283 PMC5473370

[ejsc70116-bib-0023] Frank, N. S. , S. D. Prentice , and J. P. Callaghan . 2019. “Local Dynamic Stability of the Lower Extremity in Novice and Trained Runners While Running Intraditional and Minimal Footwear.” Gait & Posture 68: 50–54. 10.1016/j.gaitpost.2018.10.034. 30458428

[ejsc70116-bib-0024] Fritz, J. , M. Knopp , H. Miles , et al. 2023. “Effects of Midsole Cushioning on Biomechanical and Physiological Performance Measures in an Elite Ultra‐Trail Runner : A Case Study.” Supplement, Footwear Science 15, no. S1: S136–S137. 10.1080/19424280.2023.2199400.

[ejsc70116-bib-0025] Fuller, J. T. , A. Amado , R. E. A. van Emmerik , et al. 2016. “The Effect of Footwear and Footfall Pattern on Running Stride Interval Long‐Range Correlations and Distributional Variability.” Gait & Posture 44: 137–142. 10.1016/j.gaitpost.2015.12.006. 27004647

[ejsc70116-bib-0026] Genitrini, M. , J. Fritz , G. Zimmermann , and H. Schwameder . 2022. “Downhill Sections Are Crucial for Performance in Trail Running Ultramarathons—A Pacing Strategy Analysis.” Journal of Functional Morphology and Kinesiology 7, no. 4: 103. 10.3390/jfmk7040103. 36412765 PMC9680470

[ejsc70116-bib-0027] Gottschall, J. S. , and R. Kram . 2005. “Ground Reaction Forces During Downhill and Uphill Running.” Journal of Biomechanics 38, no. 3: 445–452. 10.1016/j.jbiomech.2004.04.023. 15652542

[ejsc70116-bib-0028] Gullstrand, L. , K. Halvorsen , F. Tinmark , M. Eriksson , and J. Nilsson . 2009. “Measurements of Vertical Displacement in Running: A Methodological Comparison.” Gait & Posture 30, no. 1: 71–75. 10.1016/j.gaitpost.2009.03.001. 19356933

[ejsc70116-bib-0029] Hamner, S. R. , and S. L. Delp . 2013. “Muscle Contributions to fore‐aft and Vertical Body Mass Center Accelerations Over a Range of Running Speeds.” Journal of Biomechanics 46, no. 4: 780–787. 10.1016/j.jbiomech.2012.11.024. 23246045 PMC3979434

[ejsc70116-bib-0030] Hamner, S. R. , A. Seth , and S. L. Delp . 2010. “Muscle Contributions to Propulsion and Support During Running.” Journal of Biomechanics 43, no. 14: 2709–2716. 10.1016/j.jbiomech.2010.06.025. 20691972 PMC2973845

[ejsc70116-bib-0031] Hannigan, J. J. , and C. D. Pollard . 2020. “Differences in Running Biomechanics Between a Maximal, Traditional, and Minimal Running Shoe.” Journal of Science and Medicine in Sport 23, no. 1: 15–19. 10.1016/j.jsams.2019.08.008. 31501022

[ejsc70116-bib-0032] Hausdorff, J. M. , P. L. Purdon , C. K. Peng , Z. Ladin , J. Y. Wei , and A. L. Goldberger . 1996. “Fractal Dynamics of Human Gait: Stability of Long‐Range Correlations in Stride Interval Fluctuations.” Journal of Applied Physiology 80, no. 5: 1448–1457. 10.1152/jappl.1996.80.5.1448. 8727526

[ejsc70116-bib-0033] Hermsdorf, H. , N. Hofmann , and A. Keil . 2019. “Chapter 16 ‐ Alaska/Dynamicus – Human Movements in Interplay With the Environment.” In DHM and Posturography, edited by S. Scataglini and G. Paul , 187–198. Academic Press. 10.1016/B978-0-12-816713-7.00016-7.

[ejsc70116-bib-0034] Hoenig, T. , D. Hamacher , K. M. Braumann , A. Zech , and K. Hollander . 2019. “Analysis of Running Stability During 5000 m Running.” European Journal of Sport Science 19, no. 4: 413–421. 10.1080/17461391.2018.1519040. 30257130

[ejsc70116-bib-0035] Hof, A. L. 1996. “Scaling Gait Data to Body Size.” Gait & Posture 4, no. 3: 222–223. 10.1016/0966-6362(95)01057-2.

[ejsc70116-bib-0036] Honert, E. C. , and T. C. Pataky . 2021. “Timing of Gait Events Affects Whole Trajectory Analyses: A Statistical Parametric Mapping Sensitivity Analysis of Lower Limb Biomechanics.” Journal of Biomechanics 119: 110329. 10.1016/j.jbiomech.2021.110329. 33652238

[ejsc70116-bib-0037] Hoogkamer, W. 2020. “More Isn’t Always Better.” Footwear Science 12, no. 2: 75–77. 10.1080/19424280.2019.1710579.

[ejsc70116-bib-0038] Hoogkamer, W. , S. Kipp , J. H. Frank , E. M. Farina , G. Luo , and R. Kram . 2018. “A Comparison of the Energetic Cost of Running in Marathon Racing Shoes.” Sports Medicine 48, no. 4: 1009–1019. 10.1007/s40279-017-0811-2. 29143929 PMC5856879

[ejsc70116-bib-0039] Huang, M. , S. Mo , P. Pak‐Kwan Chan , Z. Y. S. Chan , J. H. Zhang‐Lea , and R. T. H. Cheung . 2022. “The Influence of Running Shoes on Familiarization Time for Treadmill Running Biomechanics Evaluation.” Sports Biomechanics: 1–14. 10.1080/14763141.2022.2046144. 35232315

[ejsc70116-bib-0040] Hunter, B. , B. Karsten , A. Greenhalgh , M. Burnley , and D. Muniz‐Pumares . 2023. “The Application of Non‐Linear Methods to Quantify Changes to Movement Dynamics During Running: A Scoping Review.” Journal of Sports Sciences 41, no. 5: 481–494. 10.1080/02640414.2023.2225014. 37330658

[ejsc70116-bib-0041] Hunter, I. , C. Bradshaw , A. McLeod , J. Ward , and T. Standifird . 2022. “Energetics and Biomechanics of Uphill, Downhill and Level Running in Highly‐Cushioned Carbon Fiber Midsole Plated Shoes.” Journal of Sports Science and Medicine 21, no. 1: 127–130. 10.52082/jssm.2022.127. 35250342 PMC8851112

[ejsc70116-bib-0042] Ji, R. , W. Y. W. Lee , X. Guan , et al. 2023. “Comparison of Plugin and Redundant Marker Sets to Analyze Gait Kinematics Between Different Populations.” BioMedical Engineering Online 22, no. 1: 122. 10.1186/s12938-023-01177-w. 38087307 PMC10717987

[ejsc70116-bib-0043] Jordan, K. , J. H. Challis , J. P. Cusumano , and K. M. Newell . 2009. “Stability and the Time‐Dependent Structure of Gait Variability in Walking and Running.” Human Movement Science 28, no. 1: 113–128. 10.1016/j.humov.2008.09.001. 19042050

[ejsc70116-bib-0044] Kettner, C. , F. Krapp , and T. Stein . 2025. “The Effects of Shoe Sole Thickness on Running Biomechanics and Economy: A Systematic Review.” Research Square. 10.21203/rs.3.rs-6526264/v1. PMC13129148 42056640

[ejsc70116-bib-0045] Kettner, C. , B. Stetter , and T. Stein . 2025a. “The Effects of Running Shoe Stack Height on Running Style and Stability During Level Running at Different Running Speeds.” Frontiers in Bioengineering and Biotechnology 13: 1–14. https://www.frontiersin.org/journals/bioengineering‐and‐biotechnology/articles/10.3389/fbioe.2025.1526752. 10.3389/fbioe.2025.1526752 PMC11885301 40059889

[ejsc70116-bib-0046] Kettner, C. , B. J. Stetter , and T. Stein . 2025b. “The Effects of Different Shoe Stack Heights and Running Speeds on Full‐Body Running Coordination: An Uncontrolled Manifold Analysis.” Journal of Biomechanics 183: 1–8. 10.1016/j.jbiomech.2025.112615. 40056729

[ejsc70116-bib-0047] King, D. L. , M. McCartney , and E. Trihy . 2019. “Initial Contact and Toe off Event Identification for Rearfoot and Non‐Rearfoot Strike Pattern Treadmill Running at Different Speeds.” Journal of Biomechanics 90: 119–122. 10.1016/j.jbiomech.2019.04.023. 31076169

[ejsc70116-bib-0048] Koegel, J. , S. Huerta , M. Gambietz , et al. 2024. “Clustering Runners ’ Response to Different Midsole Stack Heights: A Field Study.” Sensors 24, no. 4694: 1–10. 10.3390/s24144694. PMC11280980 39066091

[ejsc70116-bib-0049] Kuhman, D. J. , M. R. Paquette , S. A. Peel , and D. A. Melcher . 2016. “Comparison of Ankle Kinematics and Ground Reaction Forces Between Prospectively Injured and Uninjured Collegiate Cross Country Runners.” Human Movement Science 47: 9–15. 10.1016/j.humov.2016.01.013. 26827155

[ejsc70116-bib-0050] Kuznetsov, N. A. , and C. K. Rhea . 2017. “Power Considerations for the Application of Detrended Fluctuation Analysis in Gait Variability Studies.” PLoS One 12, no. 3: e0174144. 10.1371/journal.pone.0174144. 28323871 PMC5360325

[ejsc70116-bib-0051] Law, M. H. C. , E. M. F. Choi , S. H. Y. Law , et al. 2019. “Effects of Footwear Midsole Thickness on Running Biomechanics.” Journal of Sports Sciences 37, no. 9: 1004–1010. 10.1080/02640414.2018.1538066. 30358487

[ejsc70116-bib-0052] Leitch, J. , J. Stebbins , G. Paolini , and A. B. Zavatsky . 2011. “Identifying Gait Events Without a Force Plate During Running: A Comparison of Methods.” Gait & Posture 33, no. 1: 130–132. 10.1016/j.gaitpost.2010.06.009. 21084195

[ejsc70116-bib-0053] Lu, R. , H. Chen , J. Huang , et al. 2024. “Biomechanical Investigation of Lower Limbs During Slope Transformation Running With Different Longitudinal Bending Stiffness Shoes.” Sensors 24, no. 12: 3902. 10.3390/s24123902. 38931685 PMC11207841

[ejsc70116-bib-0054] Lussiana, T. , K. Hébert‐Losier , G. P. Millet , and L. Mourot . 2016. “Biomechanical Changes During a 50‐Minute Run in Different Footwear and on Various Slopes.” Journal of Applied Biomechanics 32, no. 1: 40–49. 10.1123/jab.2015-0108. 26367201

[ejsc70116-bib-0055] Maloney, S. J. 2018. “The Relationship Between Asymmetry and Athletic Performance: A Critical Review.” Journal of Strength & Conditioning Research 33, no. 9: 2579–2593. 10.1519/JSC.0000000000002608. 29742749

[ejsc70116-bib-0056] Mehdizadeh, S. , A. R. Arshi , and K. Davids . 2014. “Effect of Speed on Local Dynamic Stability of Locomotion Under Different Task Constraints in Running.” European Journal of Sport Science 14, no. 8: 791–798. 10.1080/17461391.2014.905986. 24720520

[ejsc70116-bib-0057] Mo, S. , F. O. Y. Lau , A. K. Y. Lok , et al. 2020. “Bilateral Asymmetry of Running Gait in Competitive, Recreational and Novice Runners at Different Speeds.” Human Movement Science 71: 102600. 10.1016/j.humov.2020.102600. 32174449

[ejsc70116-bib-0058] Möhler, F. , C. Fadillioglu , and T. Stein . 2022. “Changes in Spatiotemporal Parameters, Joint and Com Kinematics and Leg Stiffness in Novice Runners During a High‐Intensity Fatigue Protocol.” PLoS One 17, no. 4: 1–13. 10.1371/journal.pone.0265550. PMC8975020 35363776

[ejsc70116-bib-0059] Morin, J. B. , G. Dalleau , H. Kyröläinen , T. Jeannin , and A. Belli . 2005. “A Simple Method for Measuring Stiffness During Running.” Journal of Applied Biomechanics 21, no. 2: 167–180. 10.1123/jab.21.2.167. 16082017

[ejsc70116-bib-0060] Nijs, A. , M. Roerdink , and P. J. Beek . 2023. “Exploring Running Styles in the Field Through Cadence and Duty Factor Modulation.” PLoS One 18, no. 12 (December): e0295423. 10.1371/journal.pone.0295423. 38060518 PMC10703220

[ejsc70116-bib-0061] Nitschke, M. , E. Dorschky , D. Heinrich , et al. 2020. “Efficient Trajectory Optimization for Curved Running Using a 3D Musculoskeletal Model With Implicit Dynamics.” Scientific Reports 10, no. 1: 17655. 10.1038/s41598-020-73856-w. 33077752 PMC7573630

[ejsc70116-bib-0062] Ortega, J. A. , L. A. Healey , W. Swinnen , and W. Hoogkamer . 2021. “Energetics and Biomechanics of Running Footwear With Increased Longitudinal Bending Stiffness: A Narrative Review.” In Sports Medicine 51, no. 5: 873–894. Springer Science and Business Media Deutschland GmbH. 10.1007/s40279-020-01406-5. 33830444

[ejsc70116-bib-0063] Pataky, T. C. , J. Vanrenterghem , M. A. Robinson , and D. Liebl . 2019. “On the Validity of Statistical Parametric Mapping for Nonuniformly and Heterogeneously Smooth One‐Dimensional Biomechanical Data.” Journal of Biomechanics 91: 114–123. 10.1016/j.jbiomech.2019.05.018. 31155212

[ejsc70116-bib-0064] Phinyomark, A. , R. Larracy , and E. Scheme . 2020. “Fractal Analysis of Human Gait Variability Via Stride Interval Time Series.” Frontiers in Physiology 11: 333. 10.3389/fphys.2020.00333. 32351405 PMC7174763

[ejsc70116-bib-0065] Promsri, A. , S. Deedphimai , P. Promthep , and C. Champamuang . 2024. “Effects of Different Wearable Resistance Placements on Running Stability.” Sports 12, no. 2: 45. 10.3390/sports12020045. 38393265 PMC10892856

[ejsc70116-bib-0066] Raffalt, P. C. , J. A. Kent , S. R. Wurdeman , and N. Stergiou . 2019. “Selection Procedures for the Largest Lyapunov Exponent in Gait Biomechanics.” Annals of Biomedical Engineering 47, no. 4: 913–923. 10.1007/s10439-019-02216-1. 30701396 PMC6438190

[ejsc70116-bib-0067] Riazati, S. , N. Caplan , and P. R. Hayes . 2019. “The Number of Strides Required for Treadmill Running Gait Analysis is Unaffected by Either Speed or Run Duration.” Journal of Biomechanics 97: 109366. 10.1016/j.jbiomech.2019.109366. 31604569

[ejsc70116-bib-0068] Rodrigo‐Carranza, V. , F. González‐Mohíno , J. Santos del Cerro , J. Santos‐Concejero , and J. M. González‐Ravé . 2021. “Influence of Advanced Shoe Technology on the Top 100 Annual Performances in Men’s Marathon From 2015 to 2019.” Scientific Reports 11, no. 1: 22458. 10.1038/s41598-021-01807-0. 34789828 PMC8599511

[ejsc70116-bib-0069] Rodrigo‐Carranza, V. , F. González‐Mohíno , J. Santos‐Concejero , and J. M. González‐Ravé . 2020. “Influence of Shoe Mass on Performance and Running Economy in Trained Runners.” Frontiers in Physiology 11, (September): 573660. 10.3389/fphys.2020.573660. 33071828 PMC7538857

[ejsc70116-bib-0070] Rodrigo‐Carranza, V. , F. González‐Mohíno , J. Santos‐Concejero , and J. M. González‐Ravé . 2022. “The Effects of Footwear Midsole Longitudinal Bending Stiffness on Running Economy and Ground Contact Biomechanics: A Systematic Review and Meta‐Analysis.” In European Journal of Sport Science 22, no. 10: 1508–1521. Taylor and Francis Ltd. 10.1080/17461391.2021.1955014 34369282

[ejsc70116-bib-0071] Rosenstein, M. T. , J. J. Collins , and C. J. De Luca . 1993. “A Practical Method for Calculating Largest Lyapunov Exponents From Small Data Sets.” Physica D: Nonlinear Phenomena 65, no. 1: 117–134. 10.1016/0167-2789(93)90009-P.

[ejsc70116-bib-0072] Ruiz‐Alias, S. A. , D. Jaén‐Carrillo , L. E. Roche‐Seruendo , A. Pérez‐Castilla , V. M. Soto‐Hermoso , and F. García‐Pinillos . 2023. “A Review of the Potential Effects of the World Athletics Stack Height Regulation on the Footwear Function and Running Performance.” Applied Sciences 13, no. 11721: 1–11. 10.1519/JSC.0000000000004523.

[ejsc70116-bib-0073] Santuz, A. , A. Ekizos , Y. Kunimasa , K. Kijima , M. Ishikawa , and A. Arampatzis . 2020. “Lower Complexity of Motor Primitives Ensures Robust Control of High‐Speed Human Locomotion.” Heliyon 6, no. 10: e05377. 10.1016/j.heliyon.2020.e05377. 33163662 PMC7610320

[ejsc70116-bib-0074] Saunders, P. U. , D. B. Pyne , R. D. Telford , and J. A. Hawley . 2004. “Factors‐Affecting‐Running‐Economy in Trainer Distance Runners.” Sports Medicine 34, no. 7: 465–485. 10.2165/00007256-200434070-00005. 15233599

[ejsc70116-bib-0075] Schütte, K. H. , S. Seerden , R. Venter , and B. Vanwanseele . 2018. “Influence of Outdoor Running Fatigue and Medial Tibial Stress Syndrome on Accelerometer‐Based Loading and Stability.” Gait & Posture 59: 222–228. 10.1016/j.gaitpost.2017.10.021. 29080511

[ejsc70116-bib-0076] Stergiou, N. 2016. Nonlinear Analysis for Human Movement Variability. CRC Press. 10.1201/9781315370651.

[ejsc70116-bib-0077] Struzik, A. , K. Karamanidis , A. Lorimer , J. W. L. Keogh , and J. Gajewski . 2021. “Application of Leg, Vertical, and Joint Stiffness in Running Performance: A Literature Overview.” Applied Bionics and Biomechanics 2021: 1–25. 10.1155/2021/9914278. PMC8553457 34721664

[ejsc70116-bib-0078] TenBroek, T. M. , P. Rodrigues , E. C. Frederick , and J. Hamill . 2013. “Effects of Unknown Footwear Midsole Thickness on Running Kinematics Within the Initial Six Minutes of Running.” Footwear Science 5, no. 1: 27–37. 10.1080/19424280.2012.744360.

[ejsc70116-bib-0079] TenBroek, T. M. , P. A. Rodrigues , E. C. Frederick , and J. Hamill . 2014. “Midsole Thickness Affects Running Patterns in Habitual Rearfoot Strikers During a Sustained Run.” Journal of Applied Biomechanics 30, no. 4: 521–528. 10.1123/jab.2012-0224. 24615336

[ejsc70116-bib-0080] Van der Meulen, L. , S. Bonnaerens , I. Van Caekenberghe , D. De Clercq , V. Segers , and P. Fiers . 2024. “Habitual Running Style Matters: Duty Factor, and Not Stride Frequency, Relates to Loading Magnitude.” Journal of Human Kinetics 94: 37–45. 10.5114/jhk/191528. 39563764 PMC11571469

[ejsc70116-bib-0081] van Oeveren, B. T. , C. J. de Ruiter , P. J. Beek , and J. H. van Dieën . 2021. “The Biomechanics of Running and Running Styles: A Synthesis.” Sports Biomechanics 23, no. 4: 1–39. 10.1080/14763141.2021.1873411. 33663325

[ejsc70116-bib-0082] Vercruyssen, F. , M. Tartaruga , N. Horvais , and J. Brisswalter . 2016. “Effects of Footwear and Fatigue on Running Economy and Biomechanics in Trail Runners.” Medicine & Science in Sports & Exercise 48, no. 10: 1976–1984. 10.1249/MSS.0000000000000981. 27183120

[ejsc70116-bib-0083] Vicon Motion Systems Ltd . 2023. “Plug‐in Gait Reference Guide.” https://help.vicon.com/download/attachments/11378719/Plug‐in%20Gait%20Reference%20Guide.pdf.

[ejsc70116-bib-0084] Vincent, H. K. , R. Popp , O. Cicilioni , et al. 2025. “Reference Biomechanical Parameters and Natural Asymmetry Among Runners Across the Age Spectrum Without a History of running‐related Injuries.” Frontiers in Sports and Active Living 7: 1560756. 10.3389/fspor.2025.1560756. 40376183 PMC12078198

[ejsc70116-bib-0085] Wager, J. C. , and J. H. Challis . 2024. “Mechanics of the Foot and Ankle Joints During Running Using a Multi‐Segment Foot Model Compared With a Single‐Segment Model.” PLoS One 19, no. 2 (February): e0294691. 10.1371/journal.pone.0294691. 38349945 PMC10863889

[ejsc70116-bib-0086] Wallot, S. , and D. Mønster . 2018. “Calculation of Average Mutual Information (AMI) and False‐Nearest Neighbors (FNN) for the Estimation of Embedding Parameters of Multidimensional Time Series in Matlab.” Frontiers in Psychology 9, (September): 1–10. 10.3389/fpsyg.2018.01679. 30250444 PMC6139437

[ejsc70116-bib-0087] Weir, G. , H. Wyatt , R. Van Emmerik , et al. 2020. “Influence of Neutral and Stability Athletic Footwear on Lower Extremity Coordination Variability During a Prolonged Treadmill Run in Male Rearfoot Runners.” European Journal of Sport Science 20, no. 6: 776–782. 10.1080/17461391.2019.1670867. 31543009

[ejsc70116-bib-0088] Whiting, C. S. , W. Hoogkamer , and R. Kram . 2022. “Metabolic Cost of Level, Uphill, and Downhill Running in Highly Cushioned Shoes With Carbon‐Fiber Plates: Graded Running in Modern Marathon Shoes.” Journal of Sport and Health Science 11, no. 3: 303–308. 10.1016/j.jshs.2021.10.004. 34740871 PMC9189710

[ejsc70116-bib-0089] Winter, L. , P. Taylor , C. Bellenger , P. Grimshaw , and R. G. Crowther . 2024. “The Application of the Lyapunov Exponent to Analyse Human Performance: A Systematic Review.” Journal of Sports Sciences: 1–20. 10.1080/02640414.2024.2308441. 38326239

[ejsc70116-bib-0090] World Athletics Council . 2022. “Book C ‐ C2.1A Athletic Shoe Regulations.”In Book of Rules. World Athletics. https://worldathletics.org/about‐iaaf/documents/book‐of‐rules.

[ejsc70116-bib-0091] Wouda, F. J. , M. Giuberti , G. Bellusci , et al. 2018. “On the Validity of Different Motion Capture Technologies for the Analysis of Running.” In Proceedings of the IEEE RAS and EMBS International Conference on Biomedical Robotics and Biomechatronics, (August): 1175–1180. 10.1109/BIOROB.2018.8487210.

