# Supplementary material for: The Effects of Shoe Sole Thickness on Running Style and Stability During Downhill Running at Different Speeds
Source: Eur J Sport Sci. 2025 Dec 23;26(1):e70116. doi: 10.1002/ejsc.70116 (PMC12724579; doi:10.1002/ejsc.70116)
Supplement: Supplementary file 1 — Supporting Information S1. [file EJSC-26-e70116-s001.docx]

# Supplementary material


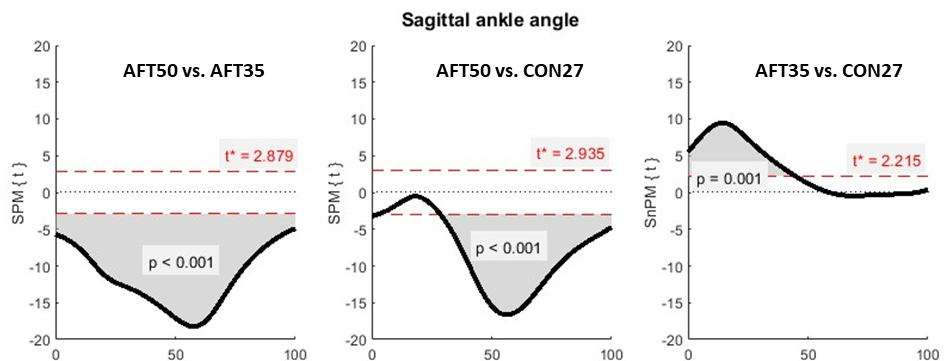


S. Figure 1: SPM t-test results of pairwise shoe comparisons at *post-hoc* for sagittal ankle angle. Significant differences are indicated by the gray areas with clustered p-values. The t* values represent the corresponding thresholds for α = 0.05. Shoe sole thicknesses are abbreviated as AFT50 (50 mm), AFT35 (35 mm), and CON27 (27 mm).


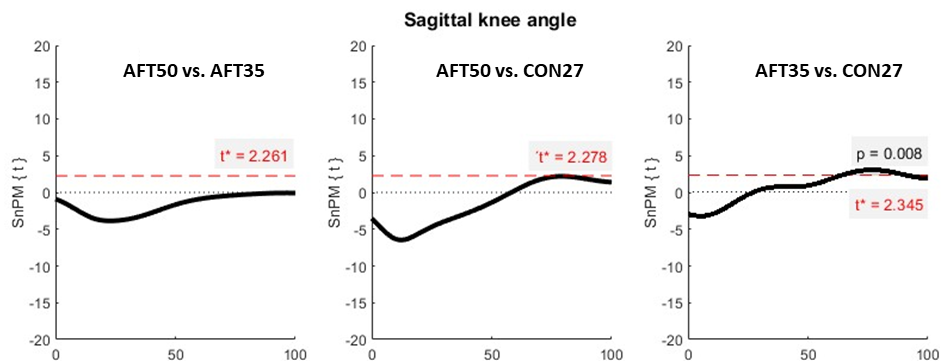


S. Figure 2: SPM t-test results of pairwise shoe comparisons at *post-hoc* for sagittal knee angle. Significant differences are indicated by the gray areas with clustered p-values. The t* values represent the corresponding thresholds for α = 0.05. Shoe sole thicknesses are abbreviated as AFT50 (50 mm), AFT35 (35 mm), and CON27 (27 mm).


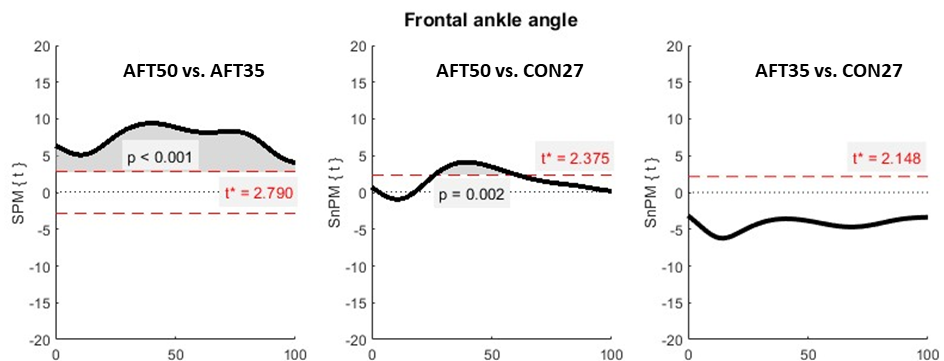


S. Figure 3: SPM t-test results of pairwise shoe comparisons at *post-hoc* for frontal ankle angle. Significant differences are indicated by the gray areas with clustered p-values. The t* values represent the corresponding thresholds for α = 0.05. Shoe sole thicknesses are abbreviated as AFT50 (50 mm), AFT35 (35 mm), and CON27 (27 mm).
